# Supplementary material for: Plasticity in striatal dopamine release is governed by release-independent depression and the dopamine transporter
Source: Nat Commun. 2019 Sep 19;10:4263. doi: 10.1038/s41467-019-12264-9 (PMC6753151; doi:10.1038/s41467-019-12264-9)
Supplement: Supplementary file 3 — Reporting Summary [file 41467_2019_12264_MOESM3_ESM.pdf]

nature research

Corresponding author(s): Stephanie Cragg

Last updated by author(s): Aug 1, 2019

Reporting Summary

Nature Research wishes to improve the reproducibility of the work that we publish. This form provides structure for consistency and transparency in reporting. For further information on Nature Research policies, see Authors & Referees and the Editorial Policy Checklist.

Statistics

For all statistical analyses, confirm that the following items are present in the figure legend, table legend, main text, or Methods section.

n/a	Confirmed	
☐	☒	The exact sample size (*n*) for each experimental group/condition, given as a discrete number and unit of measurement
☐	☒	A statement on whether measurements were taken from distinct samples or whether the same sample was measured repeatedly
☐	☒	The statistical test(s) used AND whether they are one- or two-sided<br>*Only common tests should be described solely by name; describe more complex techniques in the Methods section.*
☐	☒	A description of all covariates tested
☐	☒	A description of any assumptions or corrections, such as tests of normality and adjustment for multiple comparisons
☐	☒	A full description of the statistical parameters including central tendency (e.g. means) or other basic estimates (e.g. regression coefficient) AND variation (e.g. standard deviation) or associated estimates of uncertainty (e.g. confidence intervals)
☐	☒	For null hypothesis testing, the test statistic (e.g. *F*, *t*, *r*) with confidence intervals, effect sizes, degrees of freedom and *P* value noted<br>*Give P values as exact values whenever suitable.*
☒	☐	For Bayesian analysis, information on the choice of priors and Markov chain Monte Carlo settings
☒	☐	For hierarchical and complex designs, identification of the appropriate level for tests and full reporting of outcomes
☒	☐	Estimates of effect sizes (e.g. Cohen's *d*, Pearson's *r*), indicating how they were calculated

Our web collection on statistics for biologists contains articles on many of the points above.

Software and code

Policy information about availability of computer code

Data collection	Axoscope 10.2 and 10.5, Strathclyde Whole Cell Program, Micro-Manager 1.4, Igor Pro 6
Data analysis	As above with Microsoft Excel, GraphPad Prism 6, Matlab R2017b, Fiji 1.5

For manuscripts utilizing custom algorithms or software that are central to the research but not yet described in published literature, software must be made available to editors/reviewers. We strongly encourage code deposition in a community repository (e.g. GitHub). See the Nature Research guidelines for submitting code & software for further information.

Data

Policy information about availability of data

All manuscripts must include a data availability statement. This statement should provide the following information, where applicable:

- Accession codes, unique identifiers, or web links for publicly available datasets
- A list of figures that have associated raw data
- A description of any restrictions on data availability

The datasets generated during and/or analysed during the current study are available from the corresponding author on reasonable request.

Field-specific reporting

Please select the one below that is the best fit for your research. If you are not sure, read the appropriate sections before making your selection.

☒ Life sciences ☐ Behavioural & social sciences ☐ Ecological, evolutionary & environmental sciences

For a reference copy of the document with all sections, see nature.com/documents/nr-reporting-summary-flat.pdf

Life sciences study design

All studies must disclose on these points even when the disclosure is negative.

Sample size	No formal sample size calculation was performed. Previous experiments in our laboratory suggest that sample sizes of 3–6 animals (biological replicates) for each experimental condition are sufficient to detect drug-induced changes in dopamine release and paired-pulse ratio, and this information is balanced against the ethical requirement to use as few animals as possible.
Data exclusions	No data were excluded from this study, except in cases where dopamine release did not remain stable for the duration of the experiment due to a decline in slice viability.
Replication	The effects of changes in potassium and calcium concentration, cocaine, and 4-AP on dopamine release and plasticity have all been replicated by multiple investigators in our laboratory.
Randomization	Where possible, experimental conditions and stimulation protocols were applied in pseudorandom order, selected using a random number generator (random.org).
Blinding	Blinding was not feasible in this study, due to the need for the experimenter to apply buffers and drugs to the slice during each experiment and to subsequently match timings and calibration factors to the data during analysis.

Reporting for specific materials, systems and methods

We require information from authors about some types of materials, experimental systems and methods used in many studies. Here, indicate whether each material, system or method listed is relevant to your study. If you are not sure if a list item applies to your research, read the appropriate section before selecting a response.

Materials & experimental systems

n/a	Involved in the study	
☒	☐	Antibodies
☒	☐	Eukaryotic cell lines
☒	☐	Palaeontology
☐	☒	Animals and other organisms
☒	☐	Human research participants
☒	☐	Clinical data

Methods

n/a	Involved in the study	
☒	☐	ChIP-seq
☒	☐	Flow cytometry
☒	☐	MRI-based neuroimaging

Animals and other organisms

Policy information about studies involving animals; ARRIVE guidelines recommended for reporting animal research

Laboratory animals	Adult male C57Bl6/J mice (Jackson Laboratories), heterozygote DAT-IRES-Cre mice on a C57Bl6/J background (B6.SJL-Slc6a3tm1.1(cre)Bkmn/J, JAX stock number 006660), synapsin-III knockout (S3KO) mice, Ai95D mice (B6;129S-Gt(ROSA)26Sortm95.1(CAG-GCaMP6f)Hze/J, JAX stock number 028865) on a C57Bl6/J background.
Wild animals	*Provide details on animals observed in or captured in the field; report species, sex and age where possible. Describe how animals were caught and transported and what happened to captive animals after the study (if killed, explain why and describe method; if released, say where and when) OR state that the study did not involve wild animals.*
Field-collected samples	*For laboratory work with field-collected samples, describe all relevant parameters such as housing, maintenance, temperature, photoperiod and end-of-experiment protocol OR state that the study did not involve samples collected from the field.*
Ethics oversight	Ethical approval from the University of Oxford, and under authority of a Project Licence granted by the UK Home Office.

Note that full information on the approval of the study protocol must also be provided in the manuscript.
